# Prognostic impact of intra- and peritumoral immune cell subpopulations in head and neck squamous cell carcinomas – comprehensive analysis of the TCGA-HNSC cohort and immunohistochemical validation on 101 patients

**DOI:** 10.3389/fimmu.2023.1172768

**Published:** 2023-06-13

**Authors:** Moritz Knebel, Sandrina Körner, Jan Philipp Kühn, Silke Wemmert, Lukas Brust, Sigrun Smola, Mathias Wagner, Rainer M. Bohle, Luc G. T. Morris, Abhinav Pandey, Bernhard Schick, Maximilian Linxweiler

**Affiliations:** ^1^Department of Otorhinolaryngology, Saarland University Medical Center, Homburg, Saar, Germany; ^2^Institute of Virology, Saarland University Medical Center, Homburg, Saar, Germany; ^3^Department of General and Surgical Pathology, Saarland University Medical Center, Homburg, Saar, Germany; ^4^Department of Surgery, Memorial Sloan Kettering Cancer Center, New York City, NY, United States; ^5^Weill Cornell School of Medical Sciences, New York City, NY, United States

**Keywords:** HNSCC (head and neck squamous cell carcinoma), immunotherapy, The Cancer Genome Atlas, immunological tumor microenvironment, prognostic biomarkers

## Abstract

**Background:**

Due to the expanding role of immune checkpoint inhibition in the treatment of head and neck squamous cell carcinoma, understanding immunological processes in the tumor microevironment (TME) has strong translational importance. Though analytical methods for a comprehensive analysis of the immunological TME have constantly improved and expanded over the past years the prognostic relevance of immune cell composition in head and neck cancer TME largely remains ambiguous with most studies focusing on one or a small subset of immune cells.

**Methods:**

The overall survival (OS) of the TCGA-HNSC patient cohort comprising 513 head and neck cancer patients was correlated with a total of 29 different immune metrics including a wide spectrum of immune cell subpopulations as well as immune checkpoint receptors and cytokines using RNAseq based immune deconvolution analyses. The most significant predictors of survival among these 29 immune metrics were validated on a separate HNSCC patient cohort (n=101) using immunohistochemistry: CD3, CD20+CXCR5, CD4+CXCR5, Foxp3 and CD68.

**Results:**

Overall immune infiltration irrespective of immune cell composition showed no significant correlation with the patients’ overall survival in the TCGA-HNSC cohort. However, when focusing on different immune cell subpopulations, naïve B cells (p=0.0006), follicular T-helper cells (p<0.0001), macrophages (p=0.0042), regulatory T cells (p=0.0306), lymphocytes (p=0.0001), and cytotoxic T cells (p=0.0242) were identified as highly significant predictors of improved patient survival. Using immunohistochemical detection of these immune cells in a second independent validation cohort of 101 HNSCC patients, we confirmed the prognostic relevance of follicular T helper cells, cytotoxic T cells and lymphocytes. In multivariable analysis, HPV negativity and advanced UICC stages were identified as additional prognostic biomarkers associated with poor outcome.

**Conclusion:**

Our study highlights the prognostic relevance of the immunological tumor environment in head and neck cancer and demonstrates that a more detailed analysis of immune cell composition and immune cell subtypes is necessary to accurately prognosticate. We observed the highest prognostic relevance for lymphocytes, cytotoxic T cells, and follicular T helper cells, suggesting further investigations focusing on these specific immune cell subpopulations not only as predictors of patient prognosis but also as promising targets of new immunotherapeutic strategies.

## Introduction

Head and neck squamous cell carcinomas (HNSCC) belong to the six most common cancers worldwide, with 700,000 estimated new cases in 2018 (1). Major risk factors include chronic alcohol and tobacco consumption as well as infection of the oral/pharyngeal mucosa with high-risk human papilloma virus (HPV) (2). Patient prognosis is influenced by several clinical, molecular, and pathological factors including lymph node involvement (3), general condition (4), tumor localization (5), HPV tumor status (6), and clinical stage according to the AJCC/UICC classification (7). Over decades, the prognosis of HNSCC patients has not significantly improved, with a five year overall survival rate persisting at 50-60% (8). Even if initial treatment is successful, which in most cases encompasses a multimodal approach including surgery, radiation and/or radio-chemotherapy, more than 50% of patients develop recurrent or metastatic disease within five years after first diagnosis (9). These stalled outcomes underline the urgent need of new therapeutic approaches (10).

In this context, the introduction of immune checkpoint inhibition into the therapeutic management of various human cancer entities marked a milestone in clinical oncology and revolutionized our understanding of cancer treatment. In the field of head and neck cancer, PD1 antibodies Pembrolizumab and Nivolumab are approved for first- and second-line treatment of patients with recurrent and/or metastatic HNSCC disease (RM-HNSCC) (11–13). However, the reported objective response rates to PD1 inhibition are lower than 20% and therapy resistance is frequently observed during the treatment course. Hence, a deeper understanding of the immunological TME and its interaction with head and neck cancer cells is of great importance to improve response rates to checkpoint inhibition and refine patient selection. Studies that addressed this issue over the past years are either limited by a low number of included patients and/or investigated only one immune cell subtype or immune activity parameter, which markedly weakens the validity of obtained results and allows no relevant conclusions on a clinical situation (14). One potential approach to overcome these limitations is a sequencing of tumor genome and transcriptome in combination with advanced bioinformatic analysis. The Cancer Genome Atlas (TCGA), a project founded by the National Cancer Institute, has revealed the genomic landscape of 33 different cancers including a HNSCC cohort (15). Here, tumor tissue samples from 513 HNSCC patients were used to generate bulk genomic and transcriptomic data by next generation sequencing, which can be used for studying the tumor genome as well as TME amongst others. For a detailed characterization of the immunological tumor microenvironment several immune deconvolution protocols based on RNAseq data have been published shedding light on the complex crosstalk between tumor and immune cells (15). Through this approach, the immunological TME can be analyzed in detail, which bears the potential of identifying new prognostic and predictive biomarkers as well as therapeutic strategies.

Here, we analyzed the immunological TME of the TCGA-HNSC cohort by using bulk RNAseq data and several immune deconvolution algorithms, and correlated a total of 29 different immune metrics with overall survival. The seven most significant predictors of survival were then validated by immunohistochemistry on a second cohort of 101 HNSCC patients treated at our institution.

## Materials and methods

### TCGA data analysis and immune deconvolution

For our study, we used publicly available sequencing data from the HNSC cohort of the TCGA-project (https://portal.gdc.cancer.gov/) based on the data provided by Thorsson et al. (15). In total, the TCGA project initially included 527 HNSCC patients. After different inclusion criteria that were raised by Thorsson et al. the TCGA-HNSC cohort had to be reduced to a sample size of n=523 patient for whom immune deconvolution data were available. Due to missing clinical data including clinical follow-up and tumor localization 10 additional patients had to be excluded resulting in a final cohort of n=513 TCGA-HNSC patients that were analyzed in our study.

This TCGA-HNSC cohort comprises 309 carcinomas of the oral cavity (60%), 116 laryngeal carcinomas (22%), 78 oropharyngeal carcinomas (15%), and 10 hypopharyngeal carcinomas (2%). HPV tumor status was available for all patients using PanCancer seq data (16) with HPV positivity for 80 patients (16%). Clinical patient data including information on patient survival, TNM and clinical stage according to the AJCC/UICC classification were derived from the publicly accessible FIREBROWSE-database (http://firebrowse.org/). For assessing immune infiltration and immune activity in these tumors, several orthogonal tools based on bulk RNAseq data were applied. Single-Sample Gene Set Enrichment Analysis (ssGSEA) was developed at the Broad Institute, building on the GSEA platform (17). ssGSEA calculates enrichment scores for a sample and gene set pair, thus allowing clustering by pathways. Estimation of Stromal and Immune Cells in Malignant Tumor Tissues using Expression Data (ESTIMATE) is an algorithm based on ssGSEA developed at the MD Anderson Cancer Center in Houston (TX, USA) that integrates public databases and TCGA data (18). Differential gene expression from high and low immune cell infiltrating tumor samples were used to derive a 141-gene signature for either a “stromal score” or “immune score”, which can be combined to generate an “ESTIMATE score”. ESTIMATE immune scores for the TCGA-HNSC data set were downloaded from the MD Anderson Cancer Center website ( bioinformatics.mdanderson.org/estimate ). Immune cytolytic activity (“CYT”) score is calculated using geometric means of granzyme A (GZMA) and perforin (PRF1) gene transcripts (19). Using the aforementioned immune deconvolution methods as well as additional bulk RNAseq-based data from Thorsson et al. (15), the TCGA-HNSC patient overall survival was correlated with a total of 29 immune metrics: CD8+ T cells, CD4+ memory activated T cells, CD4+ memory resting T cells, CD4+ naive T cells, follicular helper T cells, regulatory T cells, overall lymphocyte population, overall leucocyte population, memory B cells, naive B cells, eosinophils, IFN-γ, TGF-β response, TH1 cells, TH2 cells, TH17 cells, M1 macrophages, M2 macrophages, M0 macrophages, overall macrophage population, mast cells, monocytes, neutrophils, plasma cells, activated NK cells, resting NK cells, overall dendritic cell population, activated dendritic cells, and resting dendritic cells. The underlying computational methods for defining immune cell subsets are described in detail by Thorsson et al. (15). In brief, for leucocyte fraction, overall leucocyte content was first assessed by identifying DNA methylation probes with the greatest differences between pure leucocyte cells and normal tissue, then estimating leucocyte content using a mixture model. For assessing the different immune cell subtypes within the leucocyte compartment, CIBERSORT algorithms (cell-type identification by estimating relative subsets of RNA transcripts) (20) were applied using a set of 22 immune cell reference profiles to derive a base signature matrix which can be applied to mixed samples to determine relative proportions of immune cells.

### Patients and tissue samples of the validation cohort

For the immunohistochemical analyses primary tumor tissue samples were obtained from 101 HNSCC patients. This cohort included 83% male and 17% female patients with a mean age of 63.35 years. Regarding the HPV tumor status, 82% of patients were HPV negative and 18% of the patients were tested positive with positivity being defined as both positive HPV-DNA-PCR and positive p16 immunohistochemistry. Details on the patients’ epidemiological and clinical data are shown in **Table 1**. Here, clinical stages were defined according to the 8th version of the AJCC/UICC head and neck cancer staging system (21). All patients were diagnosed and treated at the Saarland University Medical Center (Homburg, Germany). Tumor tissue of the patients was obtained from the primary tumor during diagnostic panendoscopy or surgical tumor resection. Median patient follow-up was 24 months. The Saarland Medical Association ethics review committee approved the scientific use of the patients’ tissue and clinical data (index number 218-10). All experiments were performed according to the relevant guidelines and regulations. Written informed consent was obtained from all patients.

**Table 1:** Clinical characteristics of the included patients (validation cohort).

		HNSCC patients
No. of patients		101
**sex**	male	84 (83%)
	female	17 (17%)
**Median age [years]**		63.35
**HPV status**	positive	18 (18%)
	negative	83 (82%)
**Primary tumor**	oropharynx	42 (41%)
	larynx	28 (28%)
	oral cavity	19 (19%)
	hypopharynx	12 (12%)
**UICC Stage (8th TNM version)**	I	21 (21%)
	II	18 (18%)
	III	17 (17%)
	IVa	33 (33%)
	IVb	6 (6%)
	IVc	5 (5%)
**Therapy**	surgery alone	29 (28%)
	surgery + RT	22 (22%)
	surgery + CRT	23 (23%)
	CRT alone	17 (17%)
	RT alone	1 (1%)
	RT + Cetuximab	3 (3%)
	surgery+ adjuvant RT + Cetuximab	1 (1%)
	best supportive care	4 (4%)

RT, radiotherapy; CRT, chemoradiotherapy.

### Immunohistochemistry

Immunohistochemistry was used to analyze different immune cell subpopulations in the TME of the HNSCC validation cohort. Therefore, the tumor tissue was formalin fixed, paraffin embedded and cut into 4 µm thin slices using a Leica RM 2235 rotation microtome (Leica Microsystems, Wetzlar, Germany). The slices were transferred onto Superfrost Ultra PLUS microscope slides (Menzel-Gläser, Braunschweig, Germany) and dried in an incubator overnight at 37°C. H&E staining was performed for each tissue sample according to a standard protocol for morphological control. For the immunohistochemical detection of biomarkers CD3, CD8, CD68, FoxP3, and a combination of CD4-CXCR5 and CD20-CXCR5, heat-induced epitope unmasking was performed upon deparaffinization in a rice cooker using Tris-EDTA retrieval buffer (10 mM TRIS, 1 mM EDTA, pH 9). Afterwards, unspecific protein binding was blocked by an incubation in PBS (pH 7,2) with 3% bovine serum albumine (BSA; Sigma Aldrich, St. Louis, USA) for 30 min at room temperature (RT). The slides were then incubated with the respective primary antibody. Depending on the used primary antibody incubation time ranged from 45min to 1h at RT. The final concentrations of the primary antibodies were 1:300 for CD3 (Thermo Fisher, Waltham, MA, 322 USA; clone SP7, RM-9107), 1:800 for CD8 (Abcam, Cambrigde, UK, ab4055), 1:200 for CD68 (Abcam, Cambrigde, UK, clone KP1, ab955), 1:125 for FoxP3 (Abcam, Cambrigde, UK, ab22510), 1:1500 for CXCR5 (Abcam, Cambrigde, UK, ab46218), 1:35 for CD4 (Thermo Fisher, Waltham, MA, USA; clone 4B12, MA5-12259), and 1:450 for CD20 (Abcam, Cambrigde, UK; clone L26, ab9475) in PBS/1% BSA v/v, each. Visualization was performed with streptavidin-labeled alkaline phosphatase and chromogen red using the Dako REAL Detection System Alkaline Phosphatase/RED (Dako, Glostrup, Denmark) following the manufacturer’s instructions. For the dual staining protocols, all steps as mentioned above were repeated after visualization of the first antibody sparing a second heat-induced epitope unmasking followed by incubation with the second primary antibody. Instead of using the Dako REAL Detection System RED (Dako, Glostrup, Denmark), the Dako REAL Detection System BROWN (Dako, Glostrup, Denmark) was used to visualize the second primary antibody. Finally, the slides were counterstained with hematoxylin (Sigma Aldrich) and permanently mounted with Entellan (Merck, Darmstadt, Germany). Every staining series included negative controls by omitting the primary antibody as well as appropriate positive controls (human tonsils). For the IHC experiments we used FoxP3 as surrogate marker for regulatory T cells (Treg), CD8 as surrogate marker for cytotoxic T cells, CD68 as surrogate marker for the overall macrophage population, CD3 as pan-lymphocyte marker, a combination of CD4-CXCR5 as surrogate marker for follicular T helper cells, and a combination of CD20-CXCR5 as surrogate marker for naive B cells. The stained slides were semiquantitatively assessed by a modified immune reactive score according to Remmele and Stegner (22). First, the absolute number of immune cells in the tumor and peritumoral tissue was valued from 1 to 4 (1 – no immune cells; 2 – < 25%; 3 – 25 - 50%; 4 - > 50% of tissue infiltrated with stained immune cells) and the relative number of positively stained immune cells was valued from 1 to 3 (1 - < 30%; 2 – 30 - 60%; 3 - > 60%). Both values were multiplied, resulting in a score ranging from 1 to 12. For the dual staining experiments, the positive immune cells per tissue slide were counted and valued from 1-4 (1 - 0 to 5 dual positive cells; 2 – 6 to 15 dual positive cells; 3 - 16 to 30 dual positive cells; 4 - >40 dual positive cells). To avoid a size-triggered bias, the total surface of every tissue sample as well as the area of tumor and peritumoral regions were measured using a reflected light microscope and ImageJ software. Then, the generated data on tissue size were valued from 1 to 3 (for the peritumoral region: 1- 147,2 to 293,1 mm²; 2- 31 to 126,4 mm²; 3- 0,1 to 30 mm²; for the tumor area: 1 - > 80 mm²; 2 – 16,1 to 72 mm²; 3 – 0,2 to 16 mm²). The two different values were then multiplied resulting again in a total modified IRS (mIRS) ranging from 1 to 12. Three examiners including one pathologist independently analyzed every IHC staining. All examiners were blinded for the clinical diagnosis, vitamin D level and the other examiners’ scoring.

### HPV-tumor status

HPV tumor status of the validation cohort was assessed using a combination of immunohistochemical p16 staining and HPV-DNA-PCR. Only patients with both positive p16 IHC and positive HPV-DNA-PCR were assigned a positive HPV tumor status. Regarding the significantly worse prognosis and different tumor cell biology of discordant (p16-/HPV+ or p16+/HPV-) HNSCC patients, the proof of a positive p16- and positive HPV-DNA PCR testing was mandatory for assigning positive HPV tumor status (23, 24). For HPV-DNA-PCR, DNA was extracted from fresh-frozen tumor tissue using the QIAamp DNA Blood Mini Kit (Qiagen, Hilden, Germany) following the manufacturer’s instructions. Then, HPV-DNA-PCR was performed with the LightCycler 2.0 (Roche Diagnostics, Mannheim, Germany) using GP5+/6+ primers as described previously (25). PCR amplification products were detected with SYBR Green as well as gel electrophoresis. Following an initial denaturation at 95°C for 15 min, 45 PCR cycles were performed with a denaturation at 95°C for 10 s, an annealing at 45°C for 5 s and an elongation at 72°C for 18 s. After amplification of the PCR products, a melting curve analysis was conducted with temperatures between 45°C and 95°C with a rise in temperature of 0.2°C/s. Every PCR analysis included a HPV16 positive control (Tm 79°C) and a HPV18 positive control (Tm 82°C). Additionally, the Glyceraldehyde-3-phosphat-dehydrogenase (GAPDH) gene was amplified and used as internal positive control (26).

For immunohistochemical detection of p16, the CINtec p16 histology kit (Roche Diagnostics) was used according to the manufacturer’s instructions. In brief, heat-induced epitope unmasking was performed upon deparaffinization in a rice cooker for 20 minutes using the supplied retrieval buffer. Incubation with the p16 antibody and the detection of staining signals was performed as recommended by the manufacturer. Every staining series included negative and positive controls.

### Statistical analysis

For statistical analyses, D’Agostino & Pearson omnibus normality test, Anderson-Darling test, Shapiro-Wilk test, and Kolmogorov-Smirnov test were used to determine if datasets follow a Gaussian distribution in each comparison. Gaussian distribution was only assigned if the data sample passed ≥ 2 of the aforementioned normality tests. If the data showed a normal distribution, parametric tests were performed (two-tailed unpaired t-tests, one-way ANOVA with Tukey’s correction for multiple comparisons, or Pearson correlation). If the data showed no normal distribution, non-parametric tests were applied (Mann-Whitney-U test, one-way ANOVA using Kruskal-Wallis with Dunn’s correction for multiple comparisons, or Spearman correlation). For survival analyses, a log rank test was used. Multivariant analyses were performed using a multiple logistic regression with 95% CI of the respective Odds ratio as indicated in **Supplementary Table 1**. P values < 0.05 were considered statistically significant (α=0.05).

## Results

### Prognostic relevance of immune cell infiltration and activity in the TCGA-HNSC cohort

**Figure 1:**
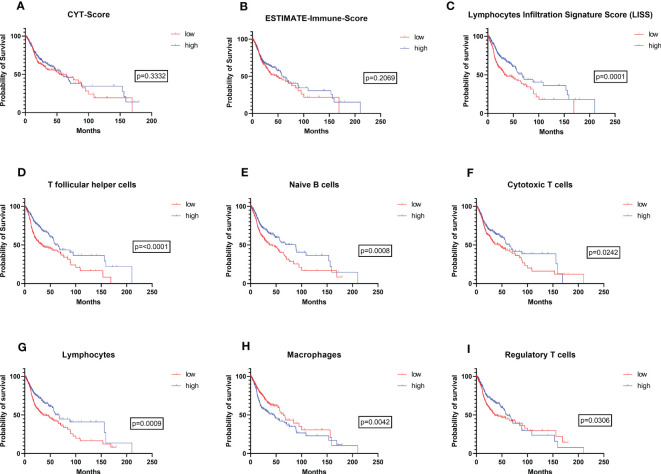
Prognostic value of immune infiltration in the TCGA-HNSC cohort. Overall survival of patients with high vs. low **(A)** CYT-Score, **(B)** ESTIMATE Immune Score, **(C)** Lymphocyte infiltration signature score (LISS), **(D)** follicular T helper cells, **(E)** naïve B cells, **(F)** cytotoxic T cells, **(G)** lymphocytes, **(H)** macrophages, and **(I)** regulatory T cells. In **(A)** to **(I)** the patients’ overall survival was analyzed using a log-rank test. P-values are indicated next to the respective graphs.

To evaluate the prognostic relevance of immune cell infiltration with different immune cell subsets as well as parameters of immune activity in the TCGA HNSC cohort (n=513), RNAseq based immune deconvolution data of 29 different immune metrics were analyzed and correlated with the patients’ overall survival: CD8+ T cells, CD4+ memory activated T cells, CD4+ memory resting T cells, CD4+ naive T cells, follicular helper T cells, regulatory T cells, overall lymphocyte population (indicated by either lymphocyte infiltration signature score (LISS; (15)) or defined as immune subpopulation based on gene expression signature scores according to (15)), overall leucocyte population, memory B cells, naive B cells, eosinophils, IFN-γ response, TGF-β response, TH1 cells, TH2 cells, TH17 cells, M1 macrophages, M2 macrophages, M0 macrophages, the overall macrophage population, mast cells, monocytes, neutrophils, plasma cells, activated NK cells, resting NK cells, the overall dendritic cell population, activated dendritic cells, and resting dendritic cells. For statistical analysis, every immune metric was divided into a high (n=257 for each immune metric) and low (n=256 for each immune metric) category, plotted around the median and correlated with the patients´ overall survival. Seven of the analyzed 29 immune metrics showed a significant correlation with the patient’s overall survival indicating a prognostic relevance for this patient cohort. Significantly improved overall survival was associated with increased follicular T helper cells (p<0.001 **Figure 1D**), Lymphocytes Infiltration Signature Score (p=0.001 **Figure 1C**), naive B cells (p=0.008 **Figure 1E**), cytotoxic T cells (p=0.0242 **Figure 1F**), lymphocytes (p=0.009 **Figure 1G**), and regulatory T cells (p=0.0306 **Figure 1**). In contrast, high infiltration of the tumor tissue with macrophages was associated with a worse outcome (p=0.0042, **Figure 1H**). As indicators of overall immune infiltration and activity, we analyzed the CYT Score (n= 482, p=0.3332) and ESTIMATE-Immune score (n=511, p=0.2069), which both showed no significant effect on overall survival (see **Figures 1A, B**). To consider the prognostic effect of HPV tumor status (see **Supplementary Figure 1**) as a potential bias in the survival analysis, we performed a multivariate analysis using logistic regression controlling for HPV tumor status in the aforementioned seven immune metrics with statistically significant relevance for patient survival. Thereby, all immune metrics excluding the naive B cell subpopulation maintained statistical significance as prognostic biomarkers (see **Supplementary Table 1**).

### Impact of HPV tumor status, tumor localization, smoking history, and patient age on tumor infiltrating lymphocytes

**Figure 2:**
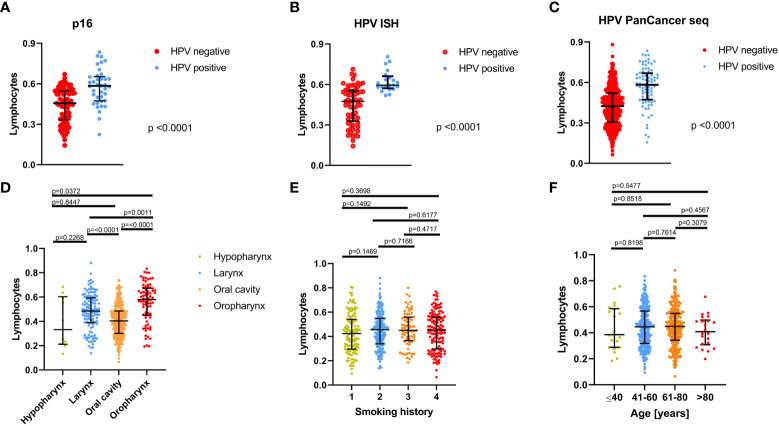
Correlation of lymphocyte infiltration with clinical and histopathological characteristics of the TCGA-HNSC patients. Correlation between lymphocyte infiltration and HPV status determined by **(A)** p16 IHC (n=111), **(B)** HPV- *in situ* hybridization (ISH; n=86), and **(C)** PanCancer HPV seq data (n=513). **(D)** Correlation of lymphocyte infiltration with tumor localization. **(E)** Correlation of lymphocyte infiltration with smoking history; here, patients were assigned to 4 categories depending on their smoking habits: patients who never smoked, (1) current smokers, (2) current reformed smokers for ≥15 years (3), and current reformed smokers for <15 years (4). **(E)** Correlation between lymphocyte infiltration and patient age. In **(A)** to **(F)**, the median is shown by a horizontal line and error bars indicate the interquartile range.

In a second step, lymphocyte infiltration based on gene expression signature scores according to Thorsson et al. (15) was used to analyze the impact of clinical and histopathological patient characteristics on the immunological TME. Thereby, HPV tumor status analyzed either by p16 IHC staining (n=111, 73 negative, 38 positive), HPV-DNA *in situ* hybridization (ISH; n= 86; 65 negative, 21 positive), or PanCancer seq data (n=513, 433 negative, 80 positive) (16) showed a strong positive correlation with lymphocyte infiltration (see **Figures 2A–C**). Additionally, primary tumor localization showed a significant impact on immune infiltration as well (p<0.0001; **Figure 2D**). Highest lymphocyte infiltration was found in oropharyngeal carcinomas (n= 78), followed by laryngeal carcinomas (n= 116), hypopharyngeal carcinomas (n=10), and oral cavity carcinomas (n=309). To exclude the patients’ smoking history (**Figure 2E**) and patient age (**Figure 2F**) as potential confounders, the lymphocyte infiltration was correlated with those clinical characteristics as well. The smoking history was defined by the patients’ smoking habits and divided into four categories: patients who never smoked (1), current smokers (2), current reformed smokers ≥15 years (3), and current reformed smokers <15 years (4). In fact, neither smoking history nor patient age showed significant effects on lymphocyte infiltration.

### Validation of the prognostic value of immune cell subsets by immunohistochemistry in an independent patient cohort

To validate the results based on RNAseq data of the TCGA-HNSC cohort, we used a second patient cohort comprising n=101 HNSCC patients (syn. validation cohort) treated at the Department of Otorhinolaryngology, Head and Neck Surgery of the Saarland University Medical Center (Homburg, Germany) for immunohistochemical analysis and correlation of those immune metrics with overall survival that showed the highest significance as prognostic biomarkers in the TCGA HNSC patients. Clinical and epidemiological data of the validation cohort patients are shown in **Table 1**.

**Figure 3:**
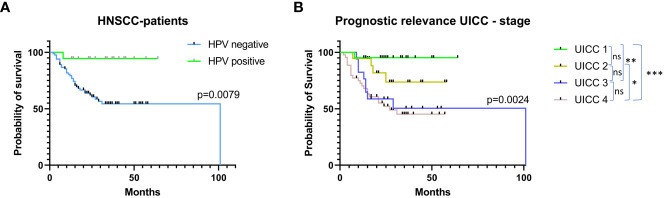
Prognostic relevance of HPV tumor status and UICC stage in the validation cohort. Patients’ overall survival **(A)** segregated by HPV tumor status. HPV positivity was defined as p16 positivity in IHC staining and positivity in HPV-DNA-PCR testing. **(B)** Overall survival depending on UICC stages according to the 8^th^ version of the TNM classification.

First, we correlated the patients’ overall survival with tumor HPV status and clinical stage according to AJCC/UICC. As shown in **Figure 3**, HPV positive patients (n=18; defined as p16 IHC + HPV-DNA-PCR positivity) showed a significantly improved outcome compared to HPV negative patients (n=83; p=0.0079). Also for clinical stages, the overall survival differed significantly depending on disease stage (p=0.0024). UICC stage one showed the best outcome with significantly longer overall survival compared to stage three (p=0.0039) and stage four (p=0.0007). Additionally, stage two patients showed a significantly improved overall survival compared to stage four patients (p=0.0436).

**Figure 4:**
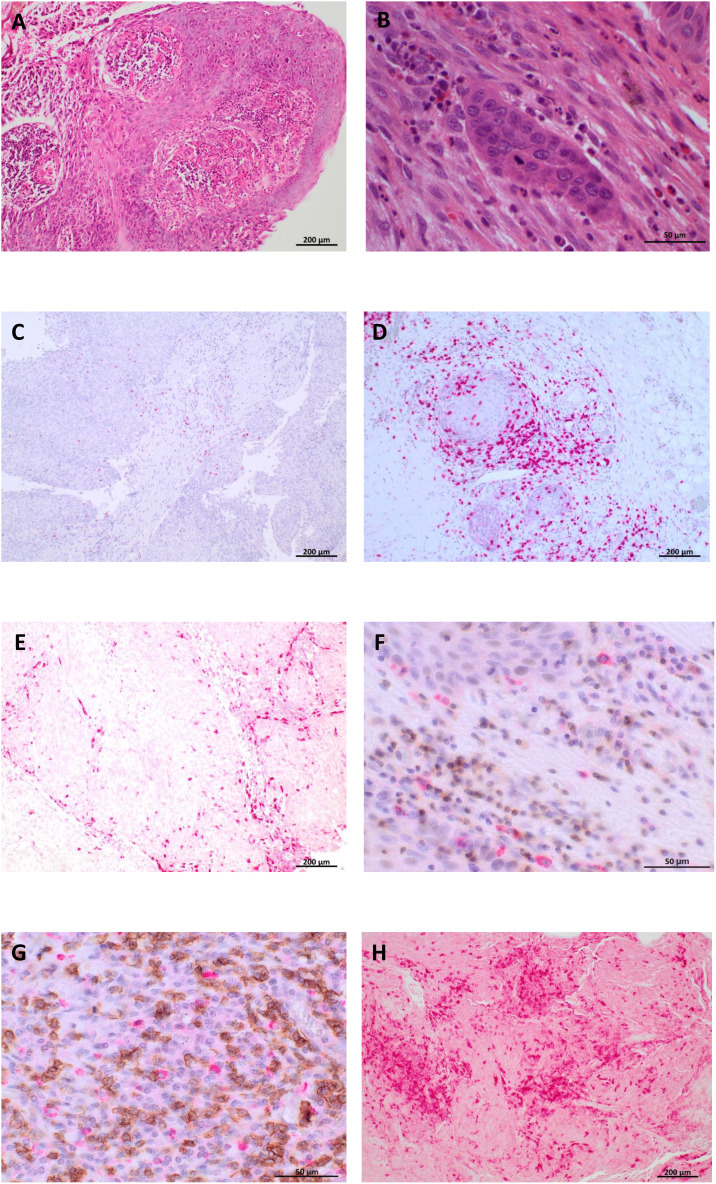
Immunohistochemical analysis of immune cell subsets in the validation cohort (syn. IHC cohort, n=101). Representative H&E as well as immunohistochemical stainings are shown for one HPV-negative hypopharyngeal cancer patient. (A+B) H&E staining in 10x **(A)** and 40x **(B)** magnification. **(C–H)** Immunohistochemical staining targeting FoxP3 (Treg cells; **(C)**), CD8 (cytotoxic T cells, **(D)**), CD68 (macrophages, **(E)**), CD4-CXCR5 (follicular helper T cells, **(F)**), CD20- CXCR5 (naive B cells, **(G)**), and CD3 (lymphocytes, **(H)**). In **(F)** and **(G)**, CXCR5 is indicated by red staining signals (FastRed) and CD4 resp. CD20 is indicated by brown staining signals (DAB).

To validate the data on prognostic significance of immune microenvironment metrics in the TCGA-HNSC cohort as shown in **Figure 1**, those immune metrics with the most relevant effect on overall survival in the TCGA HNSC cohort were analyzed and quantified by immunohistochemistry (**Figure 4**) in the validation cohort and again correlated with the patients’ outcome.

Therefore, the validation cohort was split into a high and low category defined by the mean for every single immune metric (quantified by an immunoreactive score, IRS) and correlated with the patients’ overall survival. Additionally, the peritumoral stroma and the tumor tissue itself were evaluated separately for every immune metric resulting in an intratumoral IRS and peritumoral IRS. To prevent an HPV generated bias in the survival analysis, a logistic regression analysis was performed controlling for HPV tumor status as covariate.

**Figure 5:**
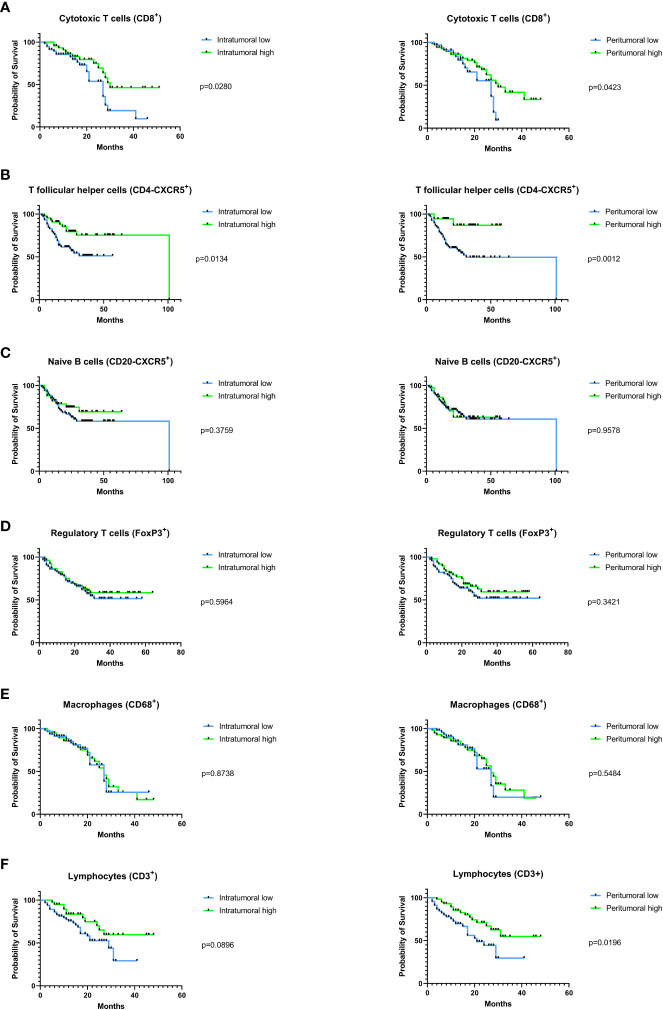
Prognostic impact of tumor infiltration by different immune cell subsets in the validation cohort. Immune cell subsets were immunohistochemically analyzed in the intra- and peritumoral compartment and were each correlated with the patients’ overall survival. Correlation between high vs. low immune infiltration with **(A)** cytotoxic T cells, **(B)** follicular T helper cells, **(C)** naive B cells, **(D)** regulatory T-cells, **(E)** macrophages, and **(F)** lymphocytes with the patients’ overall survival. In **(A)** to **(F)**, a log-rank test was used for statistical analysis, p-values are indicated next to the respective graphs.

High intratumoral (p=0.029) and peritumoral (p=0.042) infiltration with CD8^+^ cytotoxic T cells was associated with superior overall survival (**Figure 5A**). Comparable results were found for the follicular T helper cell subset (CD4-CXCR5^+^), with better OS for patients with high intratumoral (p= 0.0134) and peritumoral (p=0.0012) infiltration (**Figure 5B**). The positive prognostic effect of naive B cell (CD20-CXCR5^+^) infiltration that was found in the TCGA HNSC cohort was not confirmed in the validation cohort (intratumoral infiltration: p=0.3759, peritumoral infiltration: p=0.9579, **Figure 5C**). Similarly, we did not observe statistically significant prognostic associations with either regulatory T cells (FoxP3^+^; **Figure 5D**) or macrophages (CD68^+^; **Figure 5E**). For the overall lymphocyte population (CD3^+^), a significantly improved overall survival was observed in high vs. low peritumoral infiltration (p=0.0196, **Figure 5F**) consistent with the prognostic relevance that was shown for lymphocytes in the TCGA HNSC cohort (**Figures 1C, G**). For intratumoral lymphocyte infiltration a clear tendency towards a superior overall survival in the high infiltration group was found (p=0.0896, **Figure 5F**).

## Discussion

Head and neck cancer is one of the most immune-inflamed human tumors, but response rates to PD-1 checkpoint inhibition are modest (27) resulting in a persistently poor prognosis with five-year survival rates of 50-60% (8). Hence, the need of new biomarkers for better prognostication and therapy assignment in the rapidly evolving field of immunotherapy is evident (10, 28). Apart from the mutational landscape of tumor cells themselves, the composition of the TME crucially influences therapy response as well as prognosis of HNSCC patients (9). Against this background, 29 different biomarkers characterizing the immunological TME were analyzed in the TCGA-HNSC cohort using RNAseq based immune deconvolution data and correlated with the patients’ overall survival. The most significant predictors of survival were then immunohistochemically validated on a second cohort of 101 HNSCC patients treated at the Saarland University Medical Center. We identified 7 immune metrics including Lymphocytes Infiltration Signature Score (LISS), follicular T helper cells, naive B cells, cytotoxic T cells, lymphocytes, macrophages, and regulatory T cells associated with overall survival in the TCGA-HNSC cohort. This prognostic relevance was validated in an independent cohort, confirming the prognostic value of overall lymphocyte population, follicular T helper cells, and cytotoxic T cells.

Overall, these results are in line with recent literature in the field of head and neck immuno-oncology and support the emerging evidence that the prognostic relevance of immunological TME must be addressed in a more differentiated way (14, 29). A complex and constantly growing diversity of immune cells exert pro-inflammatory as well as immuno-suppressive effects and are engaged in a highly complex interaction with the tumor cells themselves and other cellular components of the TME (30, 31). Hence, it is not surprising that, as shown in **Figure 1**, overall immune scores that try to encompass the whole cellular network of immunological TME are not significantly affecting patient outcome. These results suggest that a more distinctive look into the different immune cell subtypes and their activity is necessary. Here, the LISS, follicular T helper cells, naive B cells, cytotoxic T cells, and lymphocytes showed the best correlation with an improved overall survival upon high infiltration levels of the tumor tissue in our study based on RNAseq immune deconvolution data in the TCGA-HNSC cohort. Inversely, macrophages showed a significantly improved overall survival upon low infiltration levels. Consistent with our study, Mandal et al. and Wondergem et al. have also examined the prognostic relevance of different immune cells in their studies including either the TCGA-HNSC cohort as well as independent further patient cohorts (10, 14). Differences between the work by Mandal et al. and our study are the number of patients that were included (Mandal et al: n=280, our study: n=513) as well the number of assessed immune metrics (Mandal et al: n=34, our study: n=29) (10). Additionally, we used the immune deconvolution data provided by Thorsson et al. as a backbone for statistically assessing the prognostic relevance of the immunological TME while Mandal et al. used their own immune deconvolution methods based on RNAseq raw data from the TCGA project and, hence, analyzed a different subset of resulting immune metrics (10, 15). The unique feature of the presented work represents an additional immunohistochemical validation of the respective TCGA data in an independent cohort of n=101 patients in order to strengthen the validity of prognostic assessment based on RNAseq data.

When we look deeper into detail for the different immune metrics, the improved overall survival upon high peri- and intratumoral lymphocyte infiltration as shown in our study is confirmed by a wide number of publications affirming a positive prognostic impact of tumor-infiltrating lymphocytes (TIL) in head and neck cancer (32, 33). Furthermore, it was shown that high TIL levels can predict better response to chemoradiation making TILs attractive not only as prognostic but also predictive biomarker in head and neck cancer (34). However, there still is a relevant lack of standardized and easily applicable methods to assess the level of TILs in HNSCC as e.g. shown by Xu et al., so that a need to establish standardized protocols for measuring TIL levels is undeniable (35).

For T follicular helper (Tfh) cells we found an improved overall survival in case of high tumor tissue infiltration representing the most significant predictor of survival among all immune metrics that were analyzed. When we look at the molecular function of Tfh cells i. e. stimulating B cells for potentiating their humoral immune responses, it is not surprising that high Tfh presence in the tumor tissue was found to be correlated with a better outcome in several solid tumor entities in recent studies (36). Additionally, there is evidence that the CD8+/follicular T helper cell crosstalk is important in shaping antitumor immune response generated by immunotherapy (37). For head and neck cancer, Cillo et al. could show a superior progression free survival in HNSCC patients with an enrichment of a CD4+ Tfh signature in the TME (38). In line with our results, these studies underline that follicular T helper cells represent not only a promising prognostic biomarker in HNSCC patients but also a potential target of new immunotherapeutic approaches due to their involvement in humoral as well as cellular antitumor immunity.

For naive B cells that also showed an association with beneficial outcome in the TCGA-HNSC cohort only few studies investigated their molecular function and prognostic importance in head and neck cancer patients. Recent studies have shown that HNSCC cells may attract naive B cells into the TME and promote their differentiation, which in turn stimulates their immunosuppressive properties within the tumor bed and thereby attenuates anti-tumor immune responses (39). On the contrary, studies in other tumor entities such as neuroblastoma could prove that naive B cells are the most consistent indicator of good prognosis and are associated with an active anti-tumor immune microenvironment (40). Hence, larger studies especially in the field of head and neck oncology will be needed to finally uncover the prognostic relevance of this immune cell subpopulation. Apart from prognostication, the different B cell phenotypes in the TME could gain importance regarding checkpoint inhibition therapy. As shown by Ruffin et al., melanoma patients who did not respond to standard of care immunotherapy i.e. anti-PD1 and/or anti-CTLA4 had significantly more naive B cells than responders, which indicates that driving naive TIL-B cells towards activated and germinal center phenotypes could be one way to complement current immunotherapeutic strategies also in head and neck cancer (41).

Literature is clearly more consistent for the prognostic role of cytotoxic T cells. A positive correlation of an improved overall survival with high tumor infiltration by cytotoxic T cells as shown in our study for the TCGA-HNSC cohort as well as the validation cohort is in line with numerous publications describing an improved progression free and overall survival upon high tumor infiltration in head and neck cancer (42–44). By additionally taking into consideration the complex interaction of cytotoxic T cells with other components of the immunological TME, e.g. the interaction of cytotoxic T cells with FoxP3+ regulatory T cells as represented by the CD8/FoxP3 ratio (43), one can even enhance the robustness and validity of CD8+ T cells as biomarker for beneficial clinical outcome in head and neck cancer (43, 45, 46).

A potential explanation for the association of increased macrophage infiltration with a worse outcome that we found in our study may be an imbalanced distribution of the pro-inflammatory M1- and immunosuppressive M2-subtypes of tumor-associated macrophages (TAM) (47). As shown by Kumar et al., a higher density of M2-like subtype of TAMs in the TME is associated with advanced T stage, increased rates of nodal positivity, presence of vascular invasion, and presence of lymphatic invasion in HNSCC (47). However, when analyzing the correlation of overall survival in the TCGA-HNSC cohort separately for M1 and M2 macrophages, no significant prognostic relevance was found for both TAM subtypes. Only in the validation cohort, high levels of M2 macrophages in the intratumoral compartment were associated with worse outcome (see **Supplementary Figure 2**). These results underline the complex function of macrophages in anti-tumor immunity which can exert immuno-stimulating as well as immunosuppressive effects depending on the molecular context and tumor entity (48).

Comparably, the prognostic relevance of regulatory T cells within head and neck cancer still remains unclear with ambiguous study result published over the past years (14). The immunological function of Tregs with repressing immune response to avoid exaggerated immune reactivity by imposing suppressive activity on CTLs and CD4 helper cells, and being involved in immune escape by secreting immunosuppressive cytokines, such as IL-10 and TGF-β, are contributing critically to an immunosuppressive milieu (9). This immunosuppressive function represents a possible explanation why we found high Treg cell infiltration being associated with worse outcome in the TCGA-HNSC cohort though this correlation could not be confirmed in the validation cohort. Mandal et al. showed that, after adjusting for HPV status, CD8+ TIS, and CD56dim NK cell infiltration, a high level of Treg cells in the TME is associated with a significantly better overall survival in HNSCC patients (10). While several other studies found an unfavorable effect of Tregs on patient outcome in HNSCC, the study published by Seminerio et al. again found an improved overall survival in case of increased levels of tumor infiltrating Tregs, which seems to support the findings by Mandal et al. as mentioned above (10, 49). Ultimately, there is no clear consensus in the literature to date regarding the prognostic significance of Tregs in HNSCC (9). Nonetheless, Treg cells are one of the largest subpopulations among all cellular components of the immunological TME in head and neck cancer, so that not only their prognostic relevance but also their suitability as therapeutic target requires further investigation (10).

From a critical point of view, one has to discuss the discrepancies in prognostic relevance of the different immune cell subtypes between the TCGA-HNSC and the IHC validation cohort. While we found consistent prognostic trends for cytotoxic T cells, follicular T helper cells, and the overall lymphocyte population, neither the naive B cells and macrophages nor the regulatory T cells could gain the same significant relevance in the validation cohort as compared to the TCGA-HNSC cohort. One potential explanation for these diverging results may be the comparably lower number of patients included in the validation cohort as well as the different methods that were used for detecting immune cell subpopulations in the TCGA-HNSC and the validation cohort. While in RNAseq analysis a variety of expression profiles and deconvolution methods can be used to identify and quantify immune cell subsets immunohistochemistry is limited to one or two biomarkers to identify a predefined immune cell type, so that there will never be a perfect match between the immune cell subpopulations identified by NGS versus IHC. On the other hand, only IHC can provide information on the localization of immune cell infiltration, which can make a relevant difference for patient prognosis as shown by our results. An additional use of NGS for characterizing the immunological TME in the validation cohort may potentially have provided deeper insights into the relevance of methodology for the aforementioned discrepancies. In future, RNAseq based analysis of the immunological TME will presumably find its way into clinical routine analysis of cancer tissue samples as NGS is getting more and more affordable though IHC still represents the present gold-standard in clinical pathology.

Taken together, we could confirm in our study that across different patient cohorts and detection methods the overall lymphocyte population, cytotoxic T cells, and follicular T helper cells seem to represent the prognostically most relevant subpopulation of tumor infiltrating leukocytes in head and neck cancer and therefore are the most promising targets of future immunotherapeutic strategies. As shown in our study, the prognostic impact of the immunological TME is highly dependent on the patient cohort as well as the applied method for identifying and quantifying different immune metrics. The fact that HNSCCs are one of the most dynamic and enthralling fields of immuno-oncological research with numerous ongoing clinical trials focusing on new immunotherapeutic approaches as well as a constantly rising knowledge on biological mechanisms of the interaction between the tumor and the immune system will further shed light on this fascinating field of cancer research. As a significant proportion of patients is still not responding to a single-agent immunotherapy, continuous effort in basic and clinical research is highly important to see if checkpoint inhibition can lead to a real paradigm shift in the treatment of head and neck cancer in the future. In this context, our study underlines that a deeper insight into and better understanding of the complex immunological TME has a great potential to facilitate better prognostication and more effective as well as precise immuno-oncological treatment of head and neck cancer.

## Data availability statement

The original contributions presented in the study are included in the article/**Supplementary Material**. Further inquiries can be directed to the corresponding author.

## Ethics statement

The studies involving human participants were reviewed and approved by The Saarland Medical Association ethics review committee (index number 218-10). The patients/participants provided their written informed consent to participate in this study.

## Author contributions

ML, SK, SW, JK, LB, and BS contributed to conception and design of the study. ML and LM analyzed the database. SS provided HPV data. MW and RB provided and analyzed tissue samples. MK performed the immunohistochemical staining and statistical analyses. MK wrote the first draft of the manuscript. ML and LM wrote sections of the manuscript. All authors contributed to the article and approved the submitted version.
