# Supplementary figures and images for: Prognostic impact of intra- and peritumoral immune cell subpopulations in head and neck squamous cell carcinomas – comprehensive analysis of the TCGA-HNSC cohort and immunohistochemical validation on 101 patients
Source: Front Immunol. 2023 Jun 13;14:1172768. doi: 10.3389/fimmu.2023.1172768 (PMC10294051; doi:10.3389/fimmu.2023.1172768)

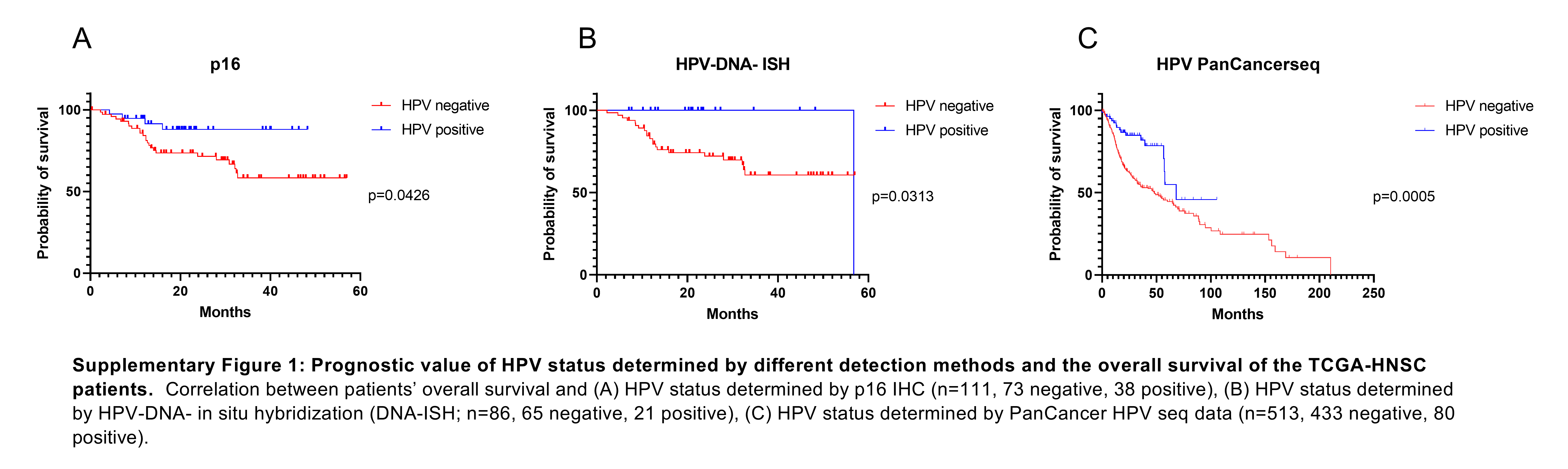

Supplement: Supplementary file 1 [file Image_1.tif]

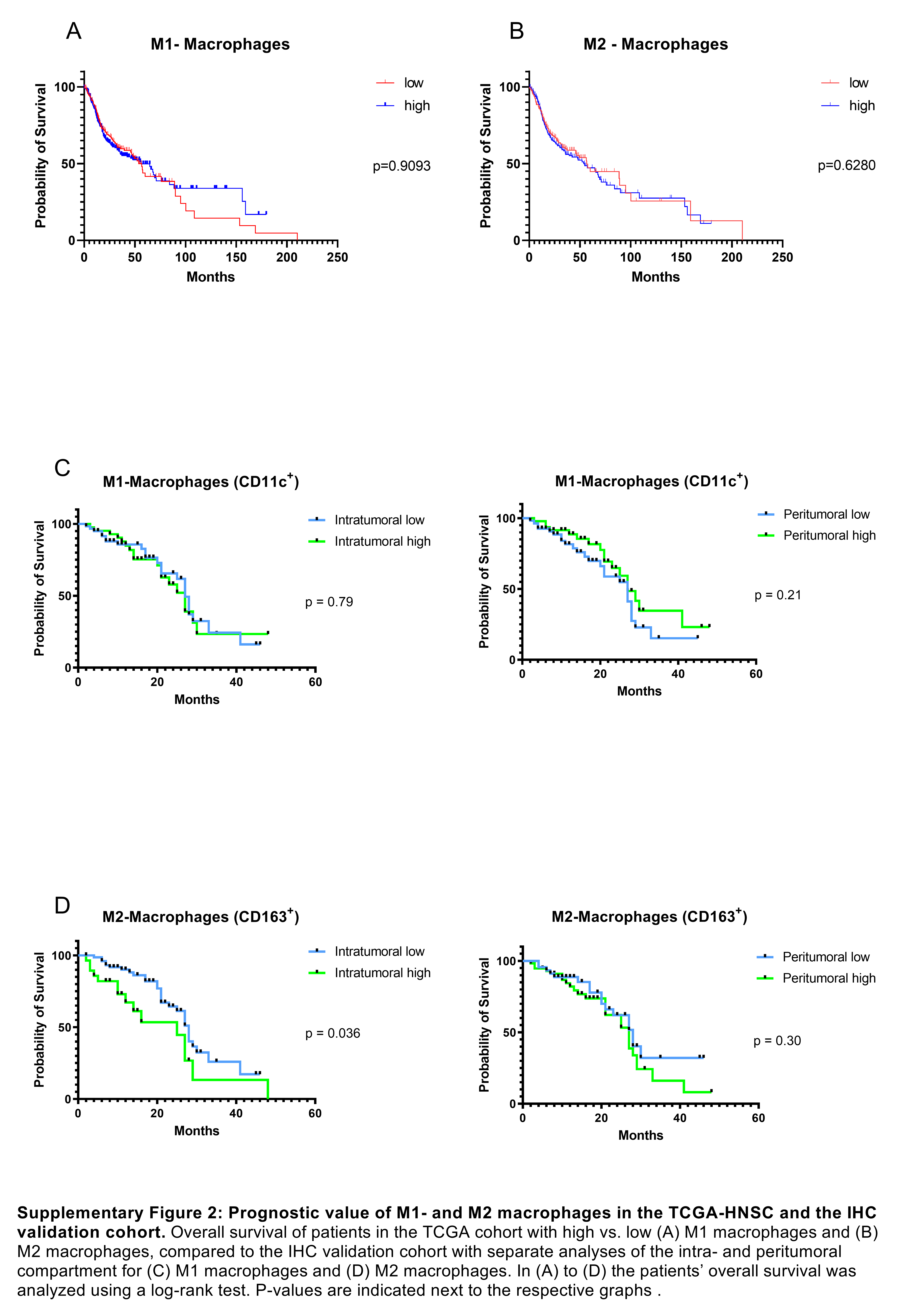

Supplement: Supplementary file 2 [file Image_2.tif]

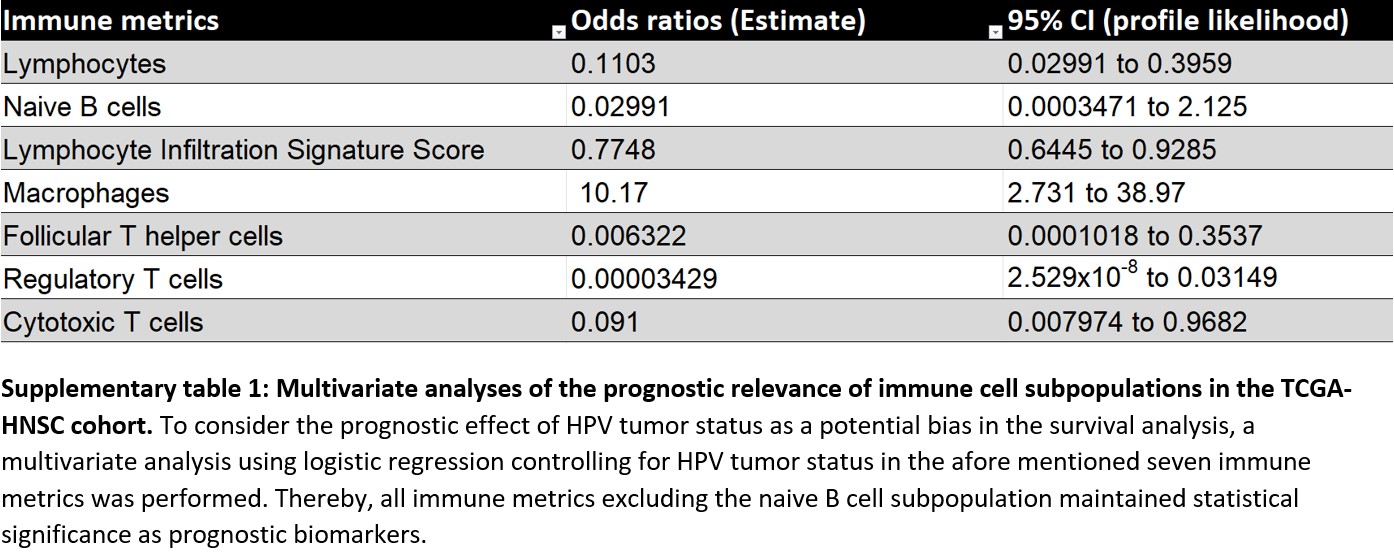

Supplement: Supplementary file 3 [file Image_3.jpeg]
